# Rosmarinic Acid Protects against Inflammation and Cardiomyocyte Apoptosis during Myocardial Ischemia/Reperfusion Injury by Activating Peroxisome Proliferator-Activated Receptor Gamma

**DOI:** 10.3389/fphar.2017.00456

**Published:** 2017-07-11

**Authors:** Jichun Han, Dong Wang, Lei Ye, Peng Li, Wenjin Hao, Xiaoyu Chen, Jun Ma, Bo Wang, Jing Shang, Defang Li, Qiusheng Zheng

**Affiliations:** ^1^School of Integrated Traditional Chinese and Western Medicine, Binzhou Medical University Yantai, China; ^2^State Key Laboratory of Natural Medicines, China Pharmaceutical University Nanjing, China; ^3^Department of Cardiac Surgery, Shandong Provincial Qianfoshan Hospital, Shandong University Jinan, China; ^4^College of Arts and Sciences, Shanxi Agricultural University Taigu, China; ^5^Key Laboratory of Xinjiang Endemic Phytomedicine Resources, Ministry of Education, School of Pharmacy, Shihezi University Shihezi, China

**Keywords:** Rosmarinic acid, PPARγ, NF-κB p65, cardio-protection, ischemia/reperfusion

## Abstract

The cardiac ischemia-reperfusion (I/R) injury greatly influences the therapeutic effect and remains an urgent challenge in clinical therapy. Polypharmacology opens a new therapeutic opportunity to design drugs with a specific target for improving the efficacy. In this study, we first forecasted that Rosmarinic acid (RosA) could be used for the treatment of cardiovascular disease using text mining, chemometric and chemogenomic methods. Consistent with the effect of the positive drug (pioglitazone, PIO), we subsequently validated that RosA pretreatment could restore the decreased cardiac hemodynamic parameters (LVDP, ± d*p*/d*t*_min_, ± d*p*/d*t*_max_ and CF), decreased the infarct size and the cardiomyocyte apoptosis in a rat model of cardiac I/R injury. Furthermore, RosA pre-treatment inhibited the levels of inflammatory cytokines (IL-6, TNF-α and CRP), up-regulated PPARγ expression and down-regulated NF-κB expression in myocardial tissue isolated from the rat model of I/R-induced myocardial injury. In addition, the effects of RosA were reversed by co-treatment with PPAR-γ inhibitor GW9662 and T0070907, respectively. These data suggest that RosA attenuates cardiac injury through activating PPARγ and down-regulating NF-κB-mediated signaling pathway, which inhibiting inflammation and cardiomyocyte apoptosis in a rat model of cardiac I/R injury.

## Introduction

Cardiovascular disease, the predominant cause of human morbidity and mortality in developed countries (Small et al., 2010). Studies have shown that ischemic heart disease is the major cause of morbidity and mortality and is forecasted to be the largest threat to human life by 2020 (Hotchkiss et al., 2013; Gunderson et al., 2014). Treatments available for myocardial infarction include restoration of the blood supply to ischemic tissue, thrombolysis, percutaneous transluminal coronary angioplasty, and coronary artery bypass surgery, as well as prevention of damage at the time of injury. However, cardiac I/R injury often occurs following surgery for acute myocardial infarction, which greatly influences the effects of therapeutics and the prognosis in patients (Biscetti et al., 2009; Huang et al., 2012). So, it’s urgent to search for an effective therapeutic method and drug to attenuate/cure the myocardial infarction.

Over the past decade, the reductionism dominated approaches have made it possible to determine the molecular and pathophysiological characteristics of the diseased heart and vasculature with the goal of developing novel diagnostic and therapeutic strategies to combat cardiovascular disease. These strategies have led to the identification of many molecules and regulatory mechanisms involved in pathological cardiovascular states, and the roles of these molecules have been studied *in vivo* using targeted pharmacological or genetic manipulation in various animal models (MacLellan et al., 2012). Nonetheless, the development of specific “one target, one drug” or euphemistically, “magic bullet” therapy is insufficient for the multigenic cardiovascular disease (Gujral et al., 2014). Therefore, polypharmacology, which focuses on designing therapeutics to target multiple receptors, has emerged as a new paradigm in drug discovery (Xie et al., 2012). Polypharmacology opens new therapeutic opportunities in the pharmaceutical industry, since designing drugs with a specific target improves the balance between efficacy and safety (Francesca and Anna, 2010). A new generation of targeted drugs from herbal medicines is currently undergoing clinical development. For example, there is emerging interest in the use of natural products, which include many targeted drugs, for the management of cardiovascular disease (Zheng et al., 2014).

Rosmarinic acid (RosA, also called α-o-caffeoyl-3,4-dihydrocyphenyl-lactic acid) is a phenolic compound isolated from a variety of Labiatae herbs (al-Sereiti et al., 1999) and has diverse immunoregulatory functions, including antioxidant (Fuhrman et al., 2000; Fadel et al., 2011), anti-inflammatory (Osakabe et al., 2004), antimicrobial (Takano et al., 2004), antiviral (Dubois et al., 2008), antirheumatic, antiallergic, antidiabetic (Petersen and Simmonds, 2003), antidepressant (Takeda et al., 2002), antiangiogenic and antitumor (Huang and Zheng, 2006). Although these various functions of RosA have been reported, very little is known regarding the multiple target action of RosA in therapeutic cardiovascular disease. Therefore, there is a motive to explore the inhibition profile of RosA on a huge panel of targets, also called “target deconvolution,” by using the computational or experimental method. However, it is still industry-wide challenge to exhaustively profile the coverage of all targets by experiment methods. The -omics (cheminformatics, proteomics, etc.) technologies are obviously the most competent approach to map drug targets reliably and efficiently (Reddy and Zhang, 2013). Well-characterized approaches, such as our previous Screening (rNTS) and reverse Network Targeting (Zheng et al., 2014), highlight the way by which these *in silico* could enable a polypharmacology approach to cardiovascular disease therapeutic. Based on the polypharmacology approach, we first predicted that RosA could be an affective component for the treatment of cardiovascular disease using text mining, chemogenomic and chemometric approaches in this study. Then, we verified the effect and mechanism of RosA in a rat model of I/R-induced myocardial injury. As far as we know, this is the first study to explore the mechanism of RosA during treatment of cardiovascular disease that used both *in silico* and *in vivo* experimental methods.

## Materials and Methods

### Target Identification

To identify the target of RosA and subsequently validate the compound-target interactions, the *in-silico* approach (Huang et al., 2014), which integrates text mining, chemometric and chemogenomic methods, was utilized in this study. First, the target of RosA was predicted using a virtual chemical fingerprint generated by the Similarity Ensemble Approach^1^. Second, a reliable omics-based LTC model with a concordance of 82.83%, sensitivity of 81.33%, and specificity of 93.62% (Yu et al., 2012) was employed for further identification and verification of RosA targets. Finally, the systematically evaluated target proteins were further screened through the Pharmacogenomics Knowledgebase^2^, Therapeutic Target Database^3^ and Comparative Toxicogenomics Database^4^ to remove noise and errors and improve accuracy.

### Comparison Potential Targets of RosA with Cardiovascular-Disease-Associated Proteins

Cardiovascular disease-associated proteins were collected by integrating the successful therapeutic protein targets and the known disease-related genes. We have compiled a high-quality, comprehensive list of cardiovascular drug-target interactions and genetic phenotype-gene associations as well as other cardiovascular disease-related information and integrated all the information into the database CVDSP (Cardiovascular Disease Systems Pharmacology). In this database, there are 268 cardiovascular disease related genes and 206 known cardiovascular targets. Combining these cardiovascular gene and target data compose a new dataset that includes 429 cardiovascular disease-related proteins (Li et al., 2014). Then, the target of RosA identified by *in-silico* approach were compared with these 429 cardiovascular-disease-associated proteins to select the potential cardiovascular disease-related target of RosA.

### The Molecular Docking Simulation

The molecular docking simulation was further performed on each bioactive compound complexed with their human targets by Surflex-Dock program combined in SYBYL 2.0 (Tripos international, United States). All the protein structures of potential target were downloaded from the RCSB protein data bank^5^, and their resolutions were carefully checked. The receptor of target proteins was optimized to remove the unrelated sub-structure. The default settings, e.g., addition of water molecules, addition of hydrogen, were used to fix the side chains of the protein structure. Meanwhile, unknown atom types were assigned and bumps were relaxed. Then, a staged minimization was performed for the protein structures using the default parameters. The docking protomol is ligand mode and the parameters are default.

### Determination of the Relative Affinity of RosA for PPARγ and PTGS2

The relative affinity of RosA for PPARγ was measured using the PolarScreen^TM^ PPAR Gamma Competitor Assay, Green (life technologies, United States) according to the manufacturer’s instructions. The Colorimetric COX (ovine) inhibitor screening assay kit (Cayman Chemical, Ann Arbor, MI, United States) was used to determine the inhibitory activities of PTGS2/COX2 (Prostaglandin G/H synthase 2).

### Test Compounds, Chemicals, and Reagents

RosA (purity 98 ≥ %) was purchased from Chengdu Must Bio-Technol Co., LTD. (Chengdu, China). PIO (purity 98 ≥ %) was purchased from Shanghai ZZBIO Co., LTD. (Shanghai, China). 1,1,3,3-tetramethoxypropane was obtained from Fluka Chemical Co. (Ronkonkoma, NY). 2,3,5-triphenyltetrazolium chloride, oxidized glutathione and reduced glutathione were purchased from Sigma Chemical Co. (St. Louis, MO, United States). All other chemicals and reagents were of analytical grade.

### Animals

Adult Sprague Dawley (SD) rats (Xinjiang Medicine University Medical Laboratory Animal Center. License Number: SCXK (xin) 2015-0013), 250–300 g, were kept in the animal facility at Shihezi University Experimental Animal Center in accord with a commercial standard mouse diet and water *ad libitum*. All rats were housed in a room maintained at a temperature of 22–25°C, relative humidity of 50–60%, and a 12-h light/12-h dark cycle. All experimental protocols in this study were performed after approval by the Institutional Animal Care and Use Committee of Shihezi University.

### Experimental Groups

Preliminary experiments of RosA treatment, using 1, 5, 10, and 20 μM doses of RosA, were performed to measure the optimal dose of RosA used in subsequent experiments. After treatment of rats with these above doses of RosA, the hemodynamic parameters in heart and the infarct size of myocardium were examined, according to the preliminary experiments data, the dose of 10 μM of RosA was used in our next experiments (Supplementary Figures 1, 2), which is in accordance with previous studies that determined that 10 μM of RosA has a cardioprotective effect (Brosková et al., 2013). Previous studies have also suggested that 5 μM of Pioglitazone (PIO), a PPARγ ligand, has a cardioprotective effect (Wynne et al., 2005), therefore, this dose of PIO was used in subsequent experiments. The rats were randomly subdivided into 12 groups: (1) Control, (2) I/R, (3) RosA, (4) PIO, (5) GW9662 (a PPAR-γ inhibitor), (6) T0070907 (a PPAR-γ inhibitor), (7) RosA+I/R, (8) PIO+I/R, (9) RosA+GW9662+I/R and (10) RosA+ T0070907+I/R, (11) PIO+GW9662+I/R and (12) PIO+T0070907+I/R. In the control group, hearts were stabilized for 15 min and subsequently perfused for 80 min. In the I/R group, hearts were subjected to 15 min of perfusion, 20 min of zero-flow global ischemia and subsequently 45 min of reperfusion after 15 min of stabilization. Hearts in the RosA, PIO, GW9662 and T0070907 groups were stabilized for 15 min, administrated with a Krebs-Henseleit (K-H) buffer containing different compounds (10 μM RosA, 5 μM PIO, 10 μM GW9662, or 10 μM T0070907) for 15 min, and then perfused for the 65 min. Hearts in the RosA+I/R, PIO+I/R, RosA+GW9662+I/R, RosA+T0070907+I/R, PIO+GW9662+ I/R, and PIO+T0070907+I/R groups were subjected to 15 min of stabilization, 15 min of different treatments with a K-H buffer solution containing different compounds (10 μM RosA, 5 μM PIO, 10 μM RosA+10 μM GW9662, 10 μM RosA+10 μM T0070907, 5 μM PIO+5 μM GW9662, or 5 μM PIO +5 μM T0070907), and then 15 min of global ischemia and 45 min of reperfusion.

### Isolation and Preparation of Rat Hearts

Sprague Dawley rats (250–300 g) were anesthetized with an intraperitoneal injection of 10% chloral hydrate (3.5 mL/kg). To prevent coagulation of the blood, 250 U/kg of heparin were administered intraperitoneally. Then, the heart was excised quickly by thoracic surgery and immediately mounted on Langendorff’s apparatus. The hearts were immersed in ice-cold K-H buffer (120 mM NaCl, 1.2 mM KH_2_PO_4_, 1.2 mM CaCl_2_, 1.2 mM MgSO_4_, 25 mM sodium acetate and 11 mM glucose, pH 7.4), equilibrated with a gas mixture comprised of 95% O_2_/5% CO_2_ at 37°C, and then incubated in a water-jacketed organ chamber at 37°C. A water-filled latex balloon combined with a Statham pressure transducer was used to insert into the left ventricular cavity through the left auricle for recording pressure.

### Determination of the Hemodynamic Parameters in Heart

A computer-based data acquisition system (PC PowerLab with Chart 5 software, 4S AD Instruments) was used to continuously monitor the hemodynamic parameters in heart. The left ventricular end-diastolic pressure (LVEDP), left ventricular systolic pressure (LVSP), left ventricular developed pressure (LVDP, LVDP = LVSP-LVEDP) and maximum rise/down velocity of the left intraventricular pressure (± d*p*/d*t_max_*) were analyzed continuously using a 4S AD Instruments biology polygraph (Powerlab, Australia). Meanwhile, a flowmeter with an in-line probe (model T106, Transonic) was used to measure the coronary flow (CF).

### Measurement of the Infarct Size of Myocardium

The hearts were subjected to 20 min of zero-flow global ischemia and then re-perfused for 45 min according to the successful ischemia and reperfusion methods previously used. For evaluation of heart tissue death, the heart was removed and washed in phosphate buffered saline (PBS). Then the hearts were frozen and stored at -20°C for 30 min. After frozen, the hearts were sliced perpendicularly along the long axis from apex to base in 1mm sections. The heart slices were incubated with 1% TTC PBS buffer (pH 7.4) at 37°C for 10–15 min, and then fixed in a 4% formaldehyde solution and subsequently photographed using a digital camera. Areas of red-stained viable tissue and white-unstained necrotic tissue were analyzed by an Image-Pro Plus 7.0 (Media Cybernetics, United States). The infarct size percentage of myocardium was calculated by the following equation:

$$\%\mathrm{Infarct}\;volume=\frac{\mathrm{Infarct}\;\mathrm{volume}}{\mathrm{Total}\;\mathrm{volume}\;\mathrm{of}\;\mathrm{slice}}\times 100$$

### Examination of Inflammatory Factors

Following the perfusions, the hearts were harvested and stored at -70°C for later analysis. The frozen tissues were weighed and then homogenized in the appropriate buffer using a micro-centrifuge tube homogenizer. After centrifugation for 10 min at 4000 rpm and 4°C, the supernatant was separated into new tube for the next analyses. C-reactive protein (CRP), interleukin-6 (IL-6) and tumor necrosis factor-α (TNF-α) were measured by spectrophotometer using Rat CRP, Rat Tumor necrosis factor alpha and Rat Interleukin 6 ELISA Kits (Tsz Biosciences, Greater Boston, MA, United States) according to the manufacturer’s instructions.

### TUNEL Assay

Terminal deoxynucleotidyl transfer-mediated dUTP nick end-labeling (TUNEL) was carried out using an *In-Situ* Cell Death Detection Kit-POD (Roche, Germany). Briefly, the heart slices were deparaffinized and rehydrated. Then the slices were treated with protease K (10mmol/L) for 15 min. After treatment, the slides were immersed in TUNEL reaction mixture in the dark at 37°C in a humidified atmosphere for 60 min. Next, the slides were incubated in converter-POD for 30 min, resulting in characteristic blue nuclear staining. Finally, the stained slices were captured and analyzed by optical microscope. To evaluate the apoptosis index of TUNEL-stained cardiac tissues, the ratio of the number of TUNEL-positive cells divided by the total number of cells was calculated as the TUNEL index (%). For each sample, eight randomly selected areas of TUNEL-stained cardiac slices were analyzed at 200× magnification and the average TUNEL index was calculated.

### Quantitative Real-Time PCR

The RNeasy Mini Kit (QIAGEN, Valencia, CA, United States) was used to isolate and purify the total RNA according to the manufacturer’s instructions. Then 1.0 μg of total RNA/sample was reverse transcribed into cDNA using the iScript cDNA synthesis kit (BIO-RosAD, Santa Rosa, CA, United States). Primers for quantitative real-time PCR (qPCR) were designed by Primer3 software and are listed in **Table 1**. Amplification of each sample was carried out using SsoFast EvaGreen Supermix (BIO-RosAD) with 10 ng of cDNA and 500 nM of each primer per reaction. Each qPCR sample was performed in triplicate in a BioRad CFX96 thermal cycler. The qPCR data was analyzed using the BioRad CFX software package. The target genes (NF-κB and PPARγ) and the internal control gene (glyceraldehyde-3-phosphate dehydrogenase, GAPDH) were amplified at equal efficiencies. The fold change of target genes was calculated using 2^-ΔΔCT^ method.

**Table 1:** The primers used for real-time PCR.

Gene		Primer sequence (5′ → 3′)
PPARγ	Forward	GGAAGACCACTCGCATTCCTT
	Reverse	GTAATCAGCAACCATTGGGTCA
NF-κB/p65	Forward	ATGGCAGACGATGATCCCTAC
	Reverse	CGGATCGAAATCCCCTCTGTT
GAPDH	Forward	TGCTGGTGCTGAGTATGTCG
	Reverse	TTGAGAGCAATGCCAGCC

### Western Blot Analysis

PPAR-γ and NF-κB protein expression levels were measured using western blot. After perfusion by the Langendorff apparatus, the same part of the rat heart was cut and collected from each sample, homogenized in the appropriate buffer (50 mM Tris-HCl, pH 7.6, 0.5% Triton X-100, 20% glycerol), and then centrifuged at 15,000 *g* for 15 min at 4°C. The supernatant was collected and boiled for 15 min to denature the proteins. The proteins in the supernatant were separated by electrophoresis on a 12% SDS polyacrylamide gel, transferred to nylon membranes by an electrophoretic transfer system, and then incubated serially with rabbit anti-rat PPAR-γ, rabbit anti-rat NF-κB and rabbit anti-rat β-actin polyclonal antibodies (Cell Signaling, Beverly, MA, United States) at 4°C overnight. The membranes were then washed with TBST buffer, and incubated with horseradish peroxidase-conjugated secondary antibody (Cell Signaling, Beverly, MA, United States). Finally, the bands were visualized using ECL-plus reagent, and the Bio-Rad Gel Doc 2000 imaging system and software were used to calculate the integrated absorbance (IA) of the bands. IA = Area × Average density. Following normalization to β-actin levels, the ratios of the IAs of PPAR-γ and NF-κB to the IA of β-actin were used to represent relative levels of activated PPAR-γ and NF-κB, respectively.

### Statistical Analysis

Data are presented as mean ± standard deviation. Statistical differences were determined using analysis of variance (ANOVA), where *P* < 0.05 was considered statistically significant. The analyses were performed using the Statistical Program for Social Sciences Software (IBM SPSS, International Business Machines Corporation, Armonk City, NY, United States).

## Results

### Target Fishing and the Relative Affinity of RosA

Combining text mining and chemogenomic prediction methods (See Materials and Methods), we gathered 32 potential protein targets for RosA (Supplementary Table 1). This high number of targets is in agreement with the polypharmacological effects of RosA. Considering these targets of RosA were identified by a proteome-wide scale. For further study, we further filtered these targets to get the specific targets. Firstly, we compared these 32 targets of RosA with the 429 cardiovascular-disease-associated protein targets that are collected from the database CVDSP (see Materials and Methods). Then we extracted seven protein targets (cardiovascular disease-related) for RosA (Supplementary Table 2). Then the molecular docking simulation was performed to analyze the interactions between RosA and the above seven protein targets. We found that the dock score of PPARγ and PTGS2 were highest (**Table 2**), so we selected PPARγ and PTGS2 as representative protein targets to validate the predicted interactions. Further studies showed that RosA activated PPARγ with EC_50_ value of 55.78 μM and inhibited PTGS2 with IC_50_ value of 169.94 μM. Given that RosA had a relatively stronger affinity for PPARγ than PTGS2 (**Table 3**), we chose the PPARγ for further pharmacological study.

**Table 2:** The dock score of seven protein targets.

Gene name	Dock score
MAPK1	7.5073
CCL2	–
AR	5.6684
PPARγ	8.9589
PTGS2	8.8219
ESR1	5.914
F2	6.8775

**Table 3:** The relative affinity of RosA for PPARγ and inhibitory activities against COX-2.

Chemical name	EC50 (mean ± SD μM)	IC50 (mean ± SD μM)
Rosmarinic acid	PPARγ	PTGS2
	55.78 ± 3.45	169.94 ± 9.18

*Values are means with their standard deviation, *n* = 6.*

### The Effect of RosA on Cardiac Function

**FIGURE 1:**
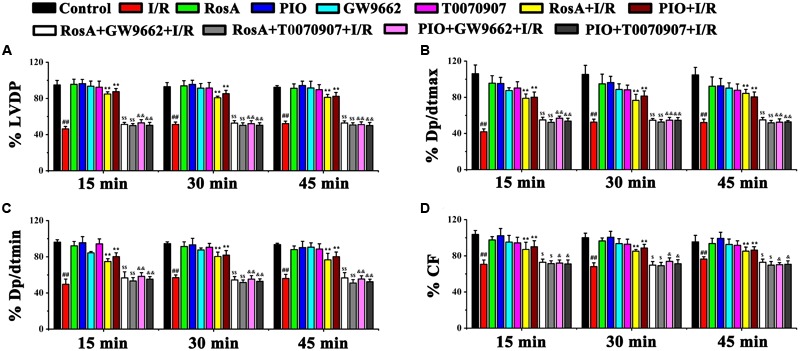
The effect of RosA on cardiac function in rat I/R model. The effect of RosA on LVDP **(A)**, *dp/dtmax* **(B)**, *dp/dtmin* **(C)**, and CF **(D)** in rat hearts. Control, healthy control mice; I/R, ischemia/reperfusion model mice; RosA, healthy mice treated with RosA; PIO, healthy mice treated with pioglitazone; GW9662, healthy mice treated with GW9662; T0070907, healthy mice treated with T0070907; RosA+I/R, I/R mice treated with RosA; PIO+I/R, I/R mice treated with PIO; RosA+GW9662+I/R, I/R mice treated with RosA plus GW9662; RosA+T0070907+I/R, I/R mice treated with RosA plus T0070907; PIO+GW9662+I/R, I/R mice treated with PIO plus GW9662; PIO+T0070907+I/R, I/R mice treated with PIO plus T0070907. Values are presented as means with their standard deviation, *n* = 6. Compared with the control group, ^#^*P* < 0.05, ^##^*P* < 0.01. Compared with the I/R group, ^∗^*P* < 0.05, ^∗∗^*P* < 0.01. Compared with the I/R+RosA group, ^$^*P* < 0.05, ^$$^*P* < 0.01. Compared with the I/R+PIO group, ^&^*P* < 0.05, ^&&^*P* < 0.01.

The dose of RosA (10 μM) used in these experiments was determined as described above. To assess whether RosA could inhibit myocardial infarct size in a PPARγ-dependent manner, inhibitors of PPARγ (GW9662 and T0070907) were used. The effects of RosA on ± d*p*/d*t*_min_, LVDP, CF and ± d*p*/d*t*_max_ were examined by a computer-based data acquisition system. Compared with the control group, these parameters were all remarkably decreased in I/R group (*P* < 0.01), showing the severe cardiac functional injury after the reperfusion (**Figures 1A–D**). After pretreatment with RosA or PIO, the hearts displayed significant functional recovery in RosA+I/R and PIO+I/R groups when compared with I/R group (*P* < 0.05), but this protective effect of RosA and PIO were both reversed by co-treatment with T0070907 and GW9662 (*P* < 0.05), respectively (**Figures 1A–D**).

### The Effect of RosA on I/R-Induced Infarct Size

**FIGURE 2:**
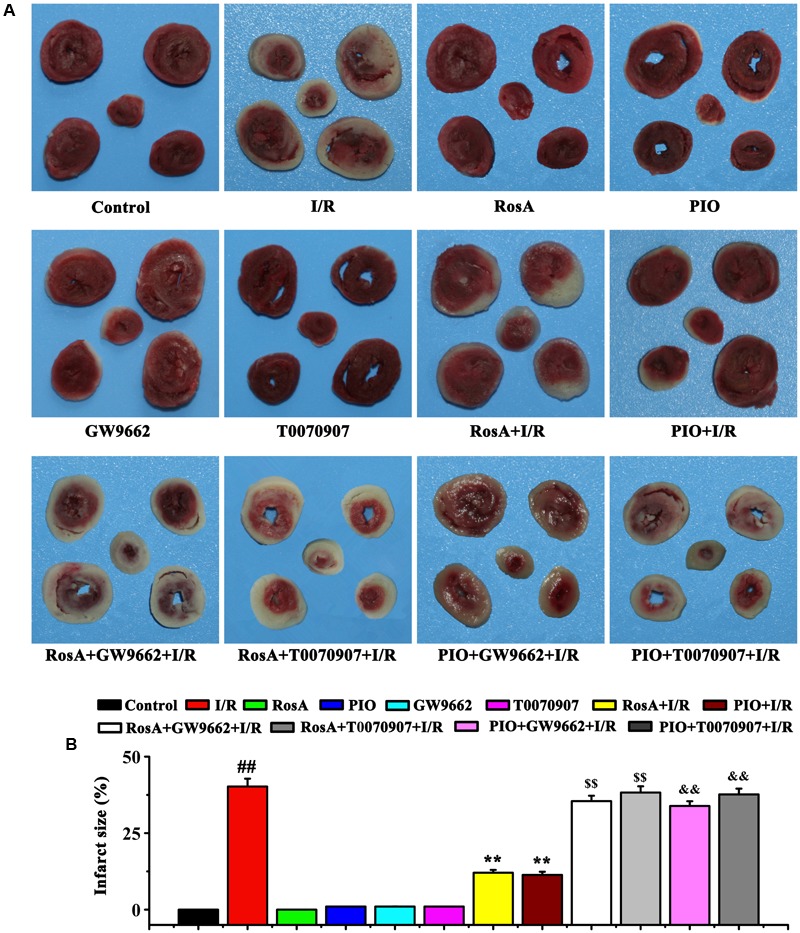
The effect of RosA on the size of I/R-induced infarcts. **(A)** The infarct size was measured by TTC staining. **(B)** Statistic analysis of the size of I/R-induced infarcts. *^##^P* < 0.01 compared to the control group; *^∗∗^P* < *0.01* compared to the I/R group. Compared with the I/R+RosA group, ^$^*P* < 0.05, ^$$^*P* < 0.01. Compared with the I/R+PIO group, ^&^*P* < 0.05, ^&&^*P* < 0.01.

Then, we examined the myocardial infarct size using TTC staining. The hearts that underwent global myocardial ischemia for 20 min followed by 45 min of reperfusion (I/R group) showed a significant increase in myocardial infarct size, pretreatment with RosA and PIO significantly inhibited this phenotype (*P* < 0.01) (**Figures 2A,B**). Compared with the RosA+I/R and PIO+I/R groups, pretreatment with GW9662 and T0070907 counteracted the effects of RosA and PIO on myocardial infarct size (**Figures 2A,B**).

### RosA Decreases the IL-6, CRP, and TNF-α Levels

**FIGURE 3:**
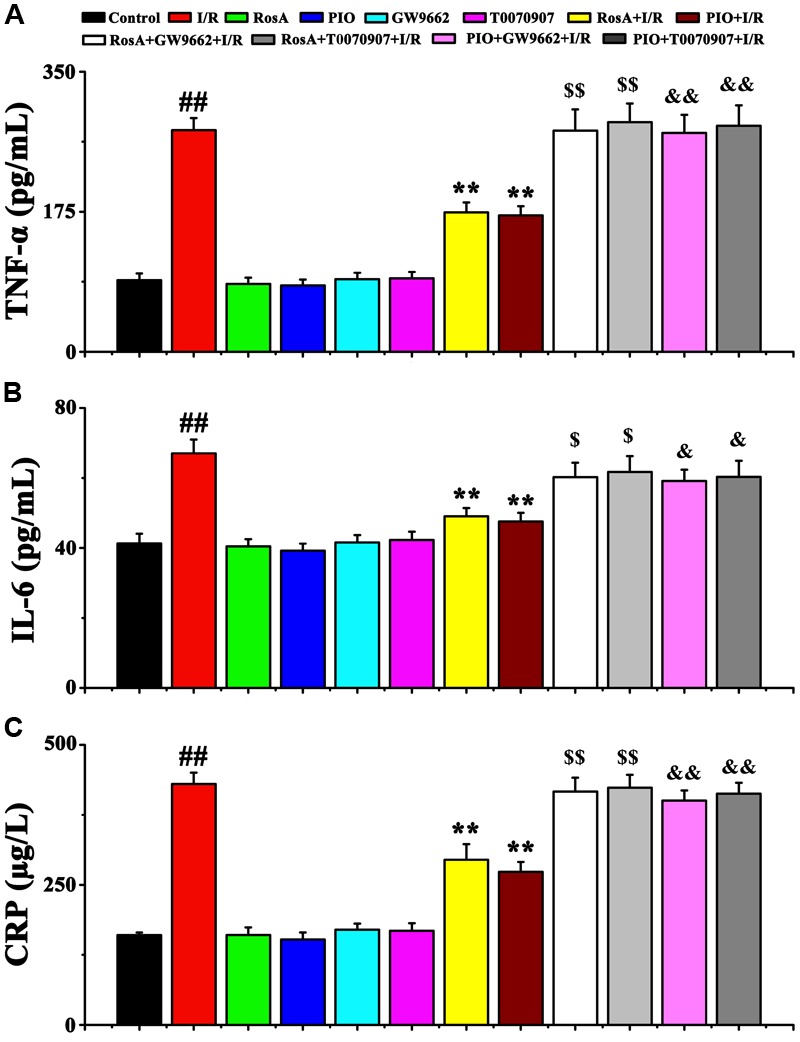
The effect of RosA on inflammatory cytokine production in rat model of cardiac I/R. Values are presented as means with their standard deviation, *n* = 6. The effect of RosA on TNF-α **(A)**, IL-6 **(B)**, and CRP **(C)** levels in I/R rat model. *^##^P* < *0.01* compared with control group; *^∗∗^P* < *0.01* compared with I/R group. Compared with the I/R+RosA group, ^$^*P* < 0.05, ^$$^*P* < 0.01. Compared with the I/R+PIO group, ^&^*P* < 0.05, ^&&^*P* < 0.01.

To characterize the possible mechanisms of RosA-mediated cardio-protection, the levels of inflammatory cytokines (e.g., TNF-α, IL-6 and CRP) in myocardial tissue were measured. Compared to the control group, the levels of IL-6, TNF-α and CRP in the I/R group significantly increased (*P* < 0.01) (**Figures 3A–C**). However, there were no significant differences in the levels of TNF-α, IL-6 and CRP between the RosA, PIO, GW9662 or T0070907 groups compared to the control. Furthermore, compared to the I/R group, the level of these 3 pro-inflammatory cytokines (TNF-α, IL-6 and CRP) were significantly decreased in the RosA+I/R and PIO+I/R groups (*P* < 0.01). Meanwhile, the effects of RosA and PIO on the levels of IL-6, CRP and TNF-α were counteracted by GW9662 and T0070907 (**Figures 3A–C**).

### RosA Decreases Cardiomyocyte Apoptosis

**FIGURE 4:**
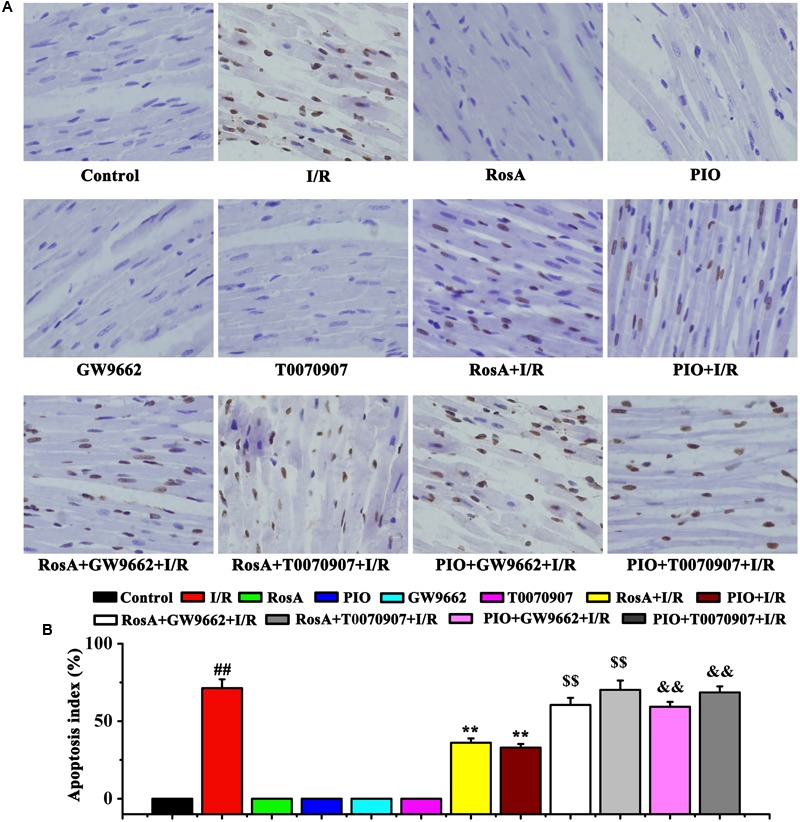
The suppression of RosA in cardiomyocyte apoptosis induced by I/R (×200). Brown staining of the nucleus is indicative of apoptosis. **(A)** The cardiomyocyte apoptosis was measured by TUNEL. **(B)** Percentage of apoptotic cells. *^##^P* < 0.01 compared to the control group; *^∗∗^P* < *0.01* compared to the I/R group. Compared with the I/R+RosA group, ^$^*P* < 0.05, ^$$^*P* < 0.01. Compared with the I/R+PIO group, ^&^*P* < 0.05, ^&&^*P* < 0.01.

As shown in **Figures 4A,B**, nuclear staining was indicative of an apoptotic cardiomyocyte nucleus, and the control group showed little or no staining. Compared to control group, the number of apoptotic cells in the I/R group dramatic increased. Compared to the I/R group, RosA+I/R and PIO+I/R groups displayed a significant reduction in the number of myocardial cell apoptosis (**Figures 4A,B**). Importantly, the effects of RosA and PIO on apoptosis were counteracted by GW9662 and T0070907, respectively (**Figures 4A,B**).

### Effect of RosA on PPAR-γ and NF-κB p65 Expression

**FIGURE 5:**
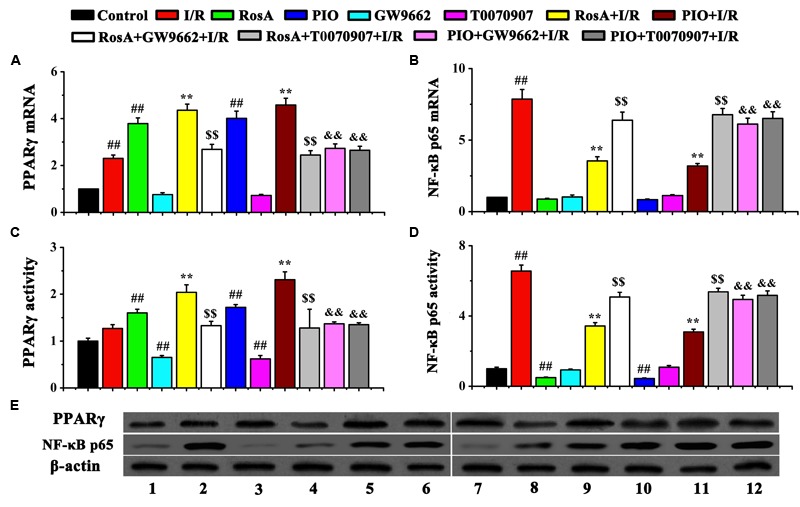
The effect of RosA on the mRNA levels and protein levels of PPARγ and NF-κB p65 in rat cardiac tissue. **(A)** The effect of RosA on the mRNA levels of PPAR-γ; **(B)** The effect of RosA on the mRNA levels of NF-κB p65; **(C)** Quantitative analysis of PPARγ protein levels; **(D)** Quantitative analysis of NF-κB p65 protein levels. **(E)** Measurement of PPARγ and NF-κB p65 protein levels in rat hearts by Western blot. (1) Control group; (2) I/R group; (3) RosA group; (4) GW9662 group; (5) RosA+I/R group; (6) RosA+GW9662+I/R group; (7) PIO group; (8) T0070907 group; (9) PIO+I/R group; (10) RosA+T0070907+I/R group; (11) PIO+GW9662+I/R group; (12) PIO+T0070907+I/R group. *^##^P* < *0.01* compared to the control group; *^∗∗^P* < *0.01* compared to the I/R group. Compared with the I/R+RosA group, ^$^*P* < 0.05, ^$$^*P* < 0.01. Compared with the I/R+PIO group, ^&^*P* < 0.05, ^&&^*P* < 0.01.

We measured the levels of PPARγ and NF-κB p65 mRNA using qPCR assay and western blot. The mRNA and protein level of the PPARγ was significantly increased in RosA+I/R and PIO+I/R groups when compared to the I/R group, while the mRNA and protein level of NF-κB p65 was remarkably decreased in the RosA+I/R and PIO+I/R groups when compared to the I/R group (**Figures 5A–E**). In addition, the effects of RosA and PIO on the expression of PPARγ and NF-κB were counteracted by GW9662 and T0070907. There was no difference in the mRNA/protein level of PPARγ and NF-κB p65 between the I/R, RosA+GW9662+I/R, RosA+T0070907+I/R, PIO+GW9662+I/R and PIO+ T0070907+I/R groups (**Figures 5A–E**).

## Discussion

The major findings in this study are that (1) RosA is calculated as a potential therapeutic molecule to attenuate/cure cardiovascular diseases using text mining, chemometric and chemogenomic methods, (2) RosA attenuates I/R-induced myocardial injury through inhibiting inflammation and cardiomyocyte apoptosis, (3) RosA inhibits inflammation and cell apoptosis through activating PPARγ and down-regulating NF-κB-mediated signaling pathway.

Drug discovery has been subjected to evolutions through the ages, moving from one drug acting on a single receptor to computational multi-target approaches. In this study, we use a new systems pharmacology method to discover drugs for cardiovascular diseases. We analyzed the hubs and the centric elements of the network to find the key targets, and we find that 32 targets are of high degree. Especially, RosA had a significant strong affinity for PPARγ, and PPARγ has been shown to play a key role in the regulation of inflammation (Taguchi et al., 2016), which could play a central role at heart injury. These provide a very good research approach for our further research.

Rosmarinic acid (also known as α-o-caffeoyl-3,4-dihydrocyphenyl-lactic acid) is a phenolic compound found in large amounts in a variety of Labiatae herbs (al-Sereiti et al., 1999) that has diverse immunoregulatory functions, including antioxidant (Fadel et al., 2011; Fialovaa et al., 2015), anti-inflammatory (Han et al., 2015), antidiabetic (Zhu et al., 2014), and antitumor (Ahamed et al., 2012). Nevertheless, very little is known regarding the mechanism of RosA as a therapeutic against cardiovascular disease. In this study, we established a rat model of I/R and observed remarkable myocardial dysfunction and significant myocardial infarct sizes, as well as significant apoptosis of cardiomyocytes. These observations agree with published reports, myocardial I/R results in heart dysfunction and apoptosis of cardiomyocytes (Oh et al., 2013; Hu N. et al., 2014). We found that treatment with RosA significantly improved the recovery of I/R-induced myocardial dysfunction, decreased I/R-induced infarct size and the rate of cardiomyocyte apoptosis. However, when PPARγ inhibitors were used, the protective effects of RosA were blocked. We also observed that PIO used as a positive control has the same protective effects as RosA.

Inflammation plays an important role in many disease, is related with increased expression of adhesive molecules in the heart and blood vessels, resulting in the infiltration of larger populations of neutrophils and monocytes/macrophages. The release of pro-inflammatory cytokines from these activated leukocytes can then in turn cause tissue damage (Varela et al., 2013). Studies have suggested that many inflammatory cytokines were released in the isolated rat heart, although there are not circulation and lymphatic system in Isolated rat heart (Glyn et al., 2003), there are many blood vessels and endothelial tissue in isolated rat hearts. In the case of ischemia and hypoxia, these vascular organizations and endothelial tissue may be release inflammatory cytokines (Guo et al., 2013; Han et al., 2014).

Several lines of evidence suggest that PPARγ may exert anti-inflammatory effects by negatively regulating the expression of pro-inflammatory genes induced during macrophage differentiation and activation (Abdelrahman et al., 2005). Along these lines, inflammation plays a key role in cardiac I/R injury, and the harmful reactions that follow these reactions include an elevated release of proinflammatory cytokines (such as CRP, TNF-α and IL-6) (Han et al., 2014; Li and Ye, 2015). Studies have demonstrated that several chemically distinct agonists of PPARγ reduce myocardial infarct size caused by regional I/R in rats (Goyal et al., 2011; Hu Q. et al., 2014). For example, treatment with telmisartan and PIO, PPARγ agonists, can substantially reduce I/R-induced myocardial infarct size. The reductions in infarct size afforded by the telmisartan and PIO correlate positively with their potency as PPARγ agonists *in vitro*. Furthermore, the protective effects of PIO are abolished by treatment with GW9662, a selective PPARγ antagonist (Wang et al., 2012; Zeng et al., 2013).

In our study, we found that RosA exhibits significant cardioprotective effects against I/R injury, decreased CRP, IL-6 and TNF-α production, as well as significantly increases PPARγ mRNA and protein expression. However, concurrent use of GW9662 or T0070907 abrogated these effects. Altogether, this data suggests that the reduction in myocardial infarct size afforded by these drugs is, at least in part, due to their ability to activate PPARγ. PPARγ also has inhibitory interactions with other transcription factors, such as NF-κB (Arab et al., 2014; Chen et al., 2014). Specifically, we found that RosA treatment significantly decreased I/R induced NF-κB mRNA and protein expression in the absence of, but not in the presence of, GW9662 or T0070907. This data suggests that NF-κB is one related target of the anti-inflammatory effects of PPARγ, and that one of the cardioprotective mechanisms of PPARγ ligands is PPARγ inhibition of NF-κB. Taken together, these results indicate that one of the mechanisms of RosA-mediated cardio-protection is through its anti-inflammatory properties, including activation of PPARγ, and, thus, inhibition of NF-κB.

**FIGURE 6:**
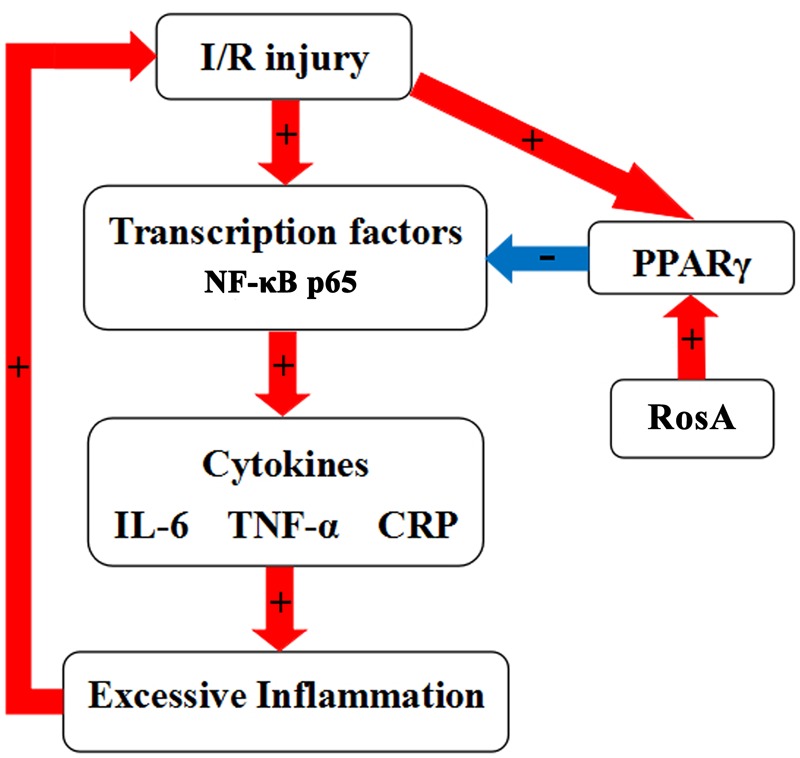
Schematic diagram of the role of the RosA in acute I/R-induced myocardial injury in isolated rat heart.

In summary, we speculate that cardiac I/R injury results in the activation of transcription factors, including NF-κB. This results in the downstream upregulation of a number of pro-inflammatory cytokines, which act in accordance with an inflammatory response. When this inflammatory response becomes unaffordable, it may subsequently aggravate the tissue injury. Cardiac tissue injury also leads to an upregulation in PPARγ expression. Our study demonstrates that activation of PPARγ by RosA inhibits the activation of NF-κB. This subsequently attenuates the production of inflammatory cytokines, thereby, reducing excessive inflammation response and tissue/organ injury. Specifically, PPARγ ligands have been shown to inhibit the levels of IL-6, TNF-α and CRP (**Figure 6**). We also found that RosA has a good antioxidative effect in isolated rat heart, indicating that one of the mechanisms of the cardio-protection of RosA was associated with its antioxidant effects (Supplementary Figure 3). Therefore, we suggest that RosA and its interactions with and effects on the cardiovascular milieu deserve an increase in experimental and clinical research. Furthermore, combining computational and experimental approaches opens up avenues of drug target discovery and characterization of the mechanisms of action for these drugs for complex diseases.

## Author Contributions

QZ and DL supervised the whole project. JH and DW performed the major research and wrote the manuscript in equal contribution. LY, PL, WH, XC, JM, and BW provided the technical support. JS provided their professional expertise.

## Conflict of Interest Statement

The authors declare that the research was conducted in the absence of any commercial or financial relationships that could be construed as a potential conflict of interest.

## Footnotes

1 http://sea.bkslab.org/

2 http://www.pharmgkb.org/

3 http://bidd.nus.edu.sg/group/ttd/ttd.asp

4 http://ctdbase.org/

5 www.pdb.org
