# Supplementary material for: Rosmarinic Acid Protects against Inflammation and Cardiomyocyte Apoptosis during Myocardial Ischemia/Reperfusion Injury by Activating Peroxisome Proliferator-Activated Receptor Gamma
Source: Front Pharmacol. 2017 Jul 11;8:456. doi: 10.3389/fphar.2017.00456 (PMC5504166; doi:10.3389/fphar.2017.00456)
Supplement: Supplementary file 1 [file Data_Sheet_1.doc]

**Rosmarinic acid protects against myocardial ischemia/reperfusion injury by activating peroxisome proliferator-activated receptor gamma**

Jichun Han^1, 2, #^, Dong Wang^3, #^, Lei Ye^1^, Peng Li^4^, Wenjin Hao^1^, Xiaoyu Chen^1^, Jun Ma^1^, Bo Wang^1^, Jing Shang^2^, Defang Li^1, #^, Qiusheng Zheng^1, 5*^

^1^Binzhou Medical University, Yantai, 264003, China;

^2^State Key Laboratory of Natural Medicines, China Pharmaceutical University, Nanjing, China,

^3^ Department of Cardiac Surgery, Shandong Provincial Qianfoshan Hospital, Shandong University, Jinan 250014, China;

^4^College of Arts and Sciences, Shanxi Agricultural University, Taigu, 030801, China;

^5^Key Laboratory of Xinjiang Endemic Phytomedicine Resources, Ministry of Education, School of Pharmacy, Shihezi University, Shihezi, 832002, China.

^#^These authors contributed equally to this work.

**^*^**Correspondence: Prof. Qiusheng Zheng ([zqsyt@sohu.com](mailto:zqsyt@sohu.com)) or Prof. Defang Li (lidefang@163.com), School of integrated traditional Chinese and Western Medicine, Binzhou Medical University, Yantai 264003, China.

**Supplementary Table 1. The information of all 32 protein targets.**

| NO. | Name |
| --- | --- |
| P1 | Mitogen-activated protein kinase 1 |
| P2 | C-C motif chemokine 2 |
| P3 | Androgen receptor |
| P4 | Peroxisome proliferator activated receptor gamma |
| P5 | Prostaglandin G/H synthase 2 |
| P6 | Estrogen receptor |
| P7 | Prothrombin |
| P8 | Complement C5 |
| P9 | C-C chemokine receptor type 3 |
| P10 | Nuclear factor of activated T-cells, cytoplasmic 3 |
| P11 | T-lymphocyte activation antigen CD80 |
| P12 | C-C motif chemokine 3 |
| P13 | Inhibitor of nuclear factor kappa-B kinase subunit beta |
| P14 | Eukaryotic translation initiation factor 6 |
| P15 | Transcription factor p65 |
| P16 | Caspase-3 |
| P17 | Signal transducer and activator of transcription 1-alpha/beta |
| P18 | G1/S-specific cyclin-D3 |
| P19 | Interleukin-2 |
| P20 | Indoleamine 2,3-dioxygenase 1 |
| P21 | Cyclin-dependent kinase inhibitor 1 |
| P22 | Alpha-synuclein |
| P23 | Interleukin-5 |
| P24 | Trypsin-1 |
| P25 | Cell division protein kinase 2 |
| P26 | Dipeptidyl peptidase IV |
| P27 | Eotaxin |
| P28 | Maltase-glucoamylase, intestinal |
| P29 | Interleukin-4 |
| P30 | Complement C3 |
| P31 | T-cell surface glycoprotein CD3 zeta chain |
| P32 | Ig gamma-1 chain C region |

**Supplementary Table 2**. Selected cardiovascular disease-related targets of RosA.

| Chemical name | Name | Gene Name |
| --- | --- | --- |
| Rosmarinic acid | Mitogen-activated protein kinase 1 | MAPK1 |
| Rosmarinic acid | C-C motif chemokine 2 | CCL2 |
| Rosmarinic acid | Androgen receptor | AR |
| Rosmarinic acid | Peroxisome proliferator activated receptor gamma | PPARγ |
| Rosmarinic acid | Prostaglandin G/H synthase 2 | PTGS2 |
| Rosmarinic acid | Estrogen receptor | ESR1 |
| Rosmarinic acid | Prothrombin | F2 |


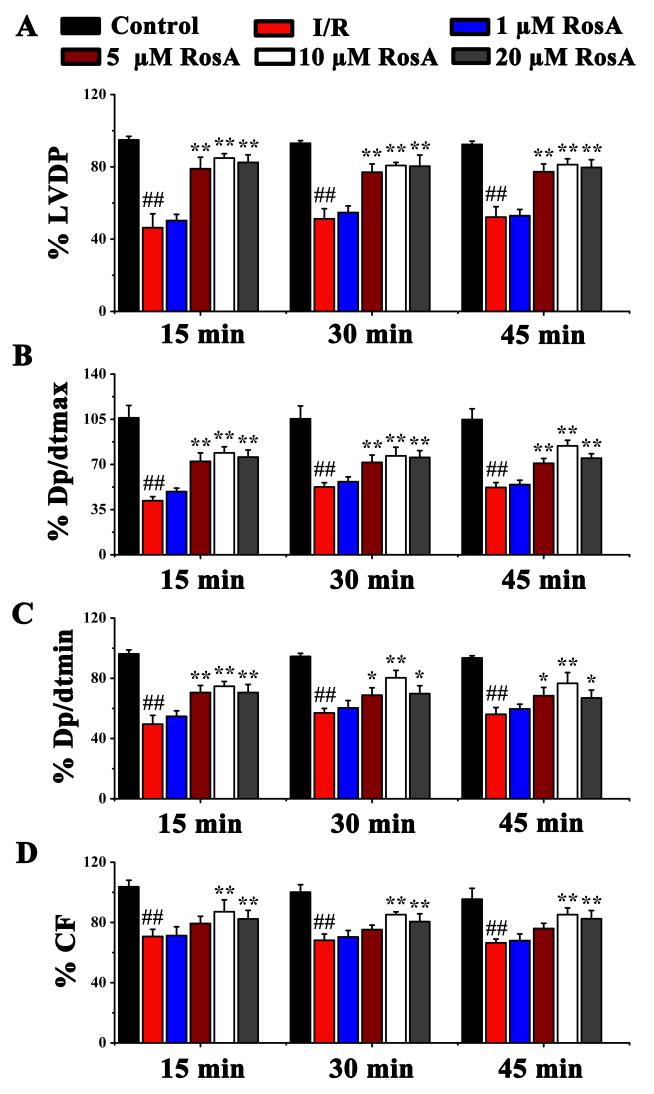


**Supplementary figure 1**. Effect of RosA on the cardiac function in rat I/R model. Effect of RosA on LVDP (A), dp/dtmax (B), dp/dtmin (C), and CF (D) in rat hearts. Note: values are presented as means with their standard deviation, n=6. *^##^P<0.01* compared to the control group; **P<0.05*, ***P<0.01* compared to the I/R group.


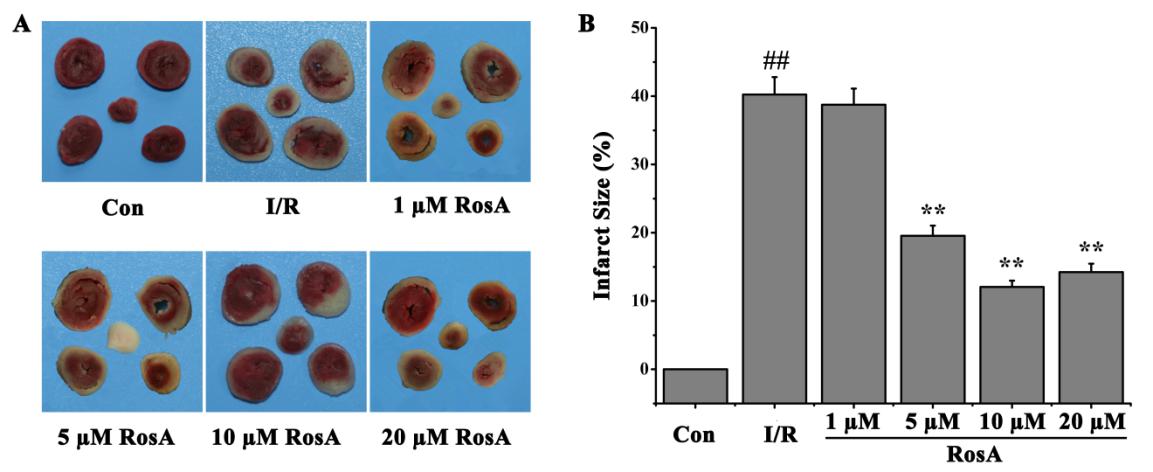


**Supplementary figure 2**. Effect of RosA on the Size of I/R-induced Infarcts in rat I/R model. (A) The infarct size was measured by TTC staining. (B) Statistic analysis of the size of I/R-induced infarcts. *^##^P<0.01* compared to the control group; **P<0.05*, ***P<0.01* compared to the I/R group.


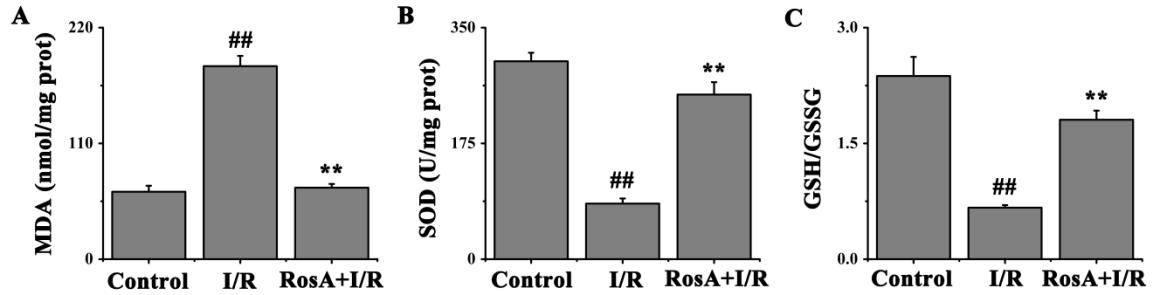


**Supplementary figure 3**. The effects of RosA on the levels of MDA, SOD and GSH/GSSG in isolated rat heart subjected to I/R. Note: values are presented as means with their standard deviation, n=6. The MDA level (A), SOD activity (B), and the ratio of GSH/GSSG (C) were determined in myocardial tissue. *^##^P<0.01* compared to the control group; **P<0.05*, ***P<0.01* compared to the I/R group.
